# Adaptation and Validation of the Diabetic Foot Ulcer Scale-Short Form in Spanish Subjects

**DOI:** 10.3390/jcm9082497

**Published:** 2020-08-03

**Authors:** Dolores Martinez-Gonzalez, Montserrat Dòria, Montserrat Martínez-Alonso, Nuria Alcubierre, Joan Valls, José Verdú-Soriano, Minerva Granado-Casas, Didac Mauricio

**Affiliations:** 1 Lleida Institute for Biomedical Research Dr. Pifarré Foundation IRBLleida, University of Lleida, Rovira Roure, 80, 25198 Lleida, Spain; lmartinezlleida@gmail.com (D.M.-G.); nurialcubierre@gmail.com (N.A.); 2 Department of Endocrinology and Nutrition, Health Sciences Research Institute and University Hospital Germans Trias i Pujol, Camí de les Escoles S/N, 08916 Badalona, Spain; montserratdoria@gmail.com; 3 Diabetic Foot Unit, University Hospital Arnau de Vilanova, Rovira Roure 80, 25198 Lleida, Spain; 4 Systems Biology and Statistical Methods for Biomedical Research, IRBLleida, University of Lleida, Rovira Roure 80, 25198 Lleida, Spain; mmartinez@irblleida.cat (M.M.-A.); jvalls@irblleida.cat (J.V.); 5 Department of Community Nursing, Preventive Medicine and Public Health and History of Science, University of Alicante, Carretera de Sant Vicent del Raspeig s/n, 03080 Alicante, Spain; pepe.verdu@ua.es; 6 Grupo Nacional de Estudio y Asesoramiento de Úlceras por Presión (GNEAUPP) Steering Committee, 26004 Logroño, Spain; 7 Centre for Biomedical Research on Diabetes and Associated Metabolic Diseases (CIBERDEM), Instituto de Salud Carlos III, 08041 Barcelona, Spain; 8 Department of Endocrinology & Nutrition, Hospital de la Santa Creu i Sant Pau, Sant Quintí, 89, 08041 Barcelona, Spain; 9 Faculty of Medicine, University of Vic (UVIC/UCC), 08500 Vic, Spain

**Keywords:** diabetic foot ulcer, type 2 diabetes, quality of life, psychometric validation, reliability, validity

## Abstract

Diabetic foot ulcer (DFU) is a chronic complication that negatively affects the quality of life (QoL) of diabetic patients. In Spain, there is no specifically designed and validated instrument to assess the QoL of patients with DFU. Our aim was to adapt the Diabetic Foot Ulcer Scale-Short Form (DFS-SF) questionnaire to a Spanish population and validate it. A prospective, observational design was used. The DFS-SF was administered by personal interview. The validated SF-36 and EQ-5D generic instruments were used as reference tools. The reliability, validity, and sensitivity to changes were assessed using standard statistical methods. A sample of 141 patients with DFU was recruited. The content validity was 3.46 on average (maximum score of 4). The internal consistency of the DFS-SF subscales showed a standardized Cronbach’s α range between 0.720 and 0.948. The DFS-SF domains showed excellent reproducibility measures (intraclass correlation coefficient from 0.77–0.92). The criterion validity was good with significant correlations between each DFS-SF subscale and its corresponding SF-36 and EQ-5D subscales (*p* < 0.001). However, the questionnaire structure was not validated (comparative fit index = 0.844, root mean square error of approximation = 0.095, and standardized root mean square residual = 0.093). The instrument showed high sensitivity to ulcer changes over time (*p* < 0.001). The adapted and validated Spanish version of the DFS-SF questionnaire has good psychometric properties and shows good sensitivity to ulcer changes, although the construct validity was not optimal. The adapted questionnaire will be a useful tool specifically to assess the QoL in subjects with diabetic foot ulcers in the clinical and research settings in Spain.

## 1. Introduction

Diabetic foot ulcer (DFU) is a condition that has a significant impact on several aspects of everyday life [1,2]. This diabetic complication usually requires multidisciplinary approaches with often intensive and prolonged treatments, which may have a substantial impact on the quality of life (QoL) [1]. Furthermore, patients with DFU are at high-risk of cardiovascular diseases in comparison with patients without this condition due to a greater burden of the disease [2]; additionally, ulceration may lead to lower-limb amputation and is associated with an increased risk of all-cause mortality [3,4]. For these reasons, it is important to assess the QoL of these patients.

QoL is a patient-reported outcome (PRO) that has become an important physical and psychological well-being measure [1]. Some observational studies reported that DFU has a negative impact on the QoL of these patients [5,6,7]. Besides, a cross-sectional study observed that patients with DFU reported a higher perception of pain related to DFU, representing a significant clinical PRO [8]. The number and severity of foot ulcers are associated with a negative health-related QoL (HRQoL) in patients with DFU compared with those patients without this diabetic complication [9]. Moreover, physical functioning and emotional role are the most affected aspects of daily life, especially in terms of leisure activity and constraints due to the treatment received [9]. Finally, while the occurrence of a minor amputation is not associated with a poorer QoL compared with patients with active DFU [10,11], the ability to maintain mobility is related to an improved HRQoL in patients with a major amputation [10].

One major limitation of the available studies is the use of different outcome instruments to assess the relationship between QoL and DFU [10]. A systematic review of the potential measures of QoL in patients with diabetes-related foot disease showed that there is no one ideal PRO for QoL assessment, so that each instrument has its limitations [1]. The use of generic instruments could underestimate the impact of the DFU on the QoL in emotional and mental domains [10]. However, disease-specific tools such as the Diabetic Foot Ulcer Scale (DFS) have shown high sensitivity to changes related to foot health or disease severity, with few confounders [1,12]. Moreover, this questionnaire offers greater information about the impact of ulcers on the QoL and is able to detect changes in DFU [1,13]. An abbreviated version of this instrument, the DFS-short form (DFS-SF), was originally designed and validated in English and afterwards adapted and validated in other languages such as Chinese, Greek, and Polish [13,14,15,16]. The available scientific evidence shows that the DFS-SF is an instrument with good psychometric properties and replicability in patients with DFU [1,13,14,15,16].

In Spain, there is no instrument specifically designed and validated to assess the impact of DFU in the QoL of diabetic patients. The aim of the study was to translate and assess the psychometric properties of the DFS-SF in a Spanish population.

## 2. Experimental Section

### 2.1. Sample and Settings

This was a prospective, observational study conducted in subjects with DFU referred from primary care and other hospital departments. Patients were treated at the Diabetic Foot Unit primarily by a podiatrist in the context of a multidisciplinary team. The DFS-SF instrument was prospectively administered at 7 days and 4, 12, and 26 weeks after the baseline assessment. The inclusion criteria were age >18 years and new-onset DFU located below the malleoli with up to 3-months duration. The exclusion criteria included cognitive deterioration, terminal illnesses, and hospitalization. A trained researcher (D.M.-G.) performed individual interviews with all of the patients and reviewed each of their clinical records carefully to collect the following clinical variables: Age; sex; educational level; smoking habit; type of diabetes, disease duration, and diabetes-specific therapy; the presence of hypertension and dyslipidemia if they were diagnosed with the disease or were receiving for medication for any of these conditions; the presence of cardiovascular diseases (i.e., cerebrovascular disease, peripheral artery disease, and ischemic heart disease); the diagnose of diabetic retinopathy; dialysis (including both hemodialysis and peritoneal dialysis); and the use of antiplatelet agents. Glycated hemoglobin (HbA1c) was collected using the most recent value within the previous 6 months. Diabetic nephropathy was defined as an estimated glomerular filtration rate (eGFR) below 60 mL/min and/or an albumin-to-creatinine urine ratio over 30 mg/g. The diagnosis of DFU was defined according to the consensus of the International Working Group on the Diabetic Foot (IWGDF) [17]. A detailed foot examination was performed to determine the presence of previous lower-limb amputations (minor o major), deformities, or the diagnosis of Charcot foot disease and to assess the local ulcer characteristics [18,19,20]. Peripheral neuropathy was assessed using a biothensiometer (Me. Te. Da. Srl., IT) using standard procedures, as described previously [18]. Peripheral arterial disease was evaluated through the ankle-brachial index (ABI) value and classified as normal (between 0.91 and 1.30), moderate ischemia (between 0.41 and 0.90), severe ischemia (between 0 and 0.40), and non-compressible due to the presence of calcification (over 1.30) [21]. In those patients with an ABI value of 1.30 or greater, the pedal or posterior tibial pulse were explored; the diagnosis of peripheral arterial disease was defined by the presence of a non-palpable pulses. The ulcer was classified as neuropathic, ischemic and neuroischemic according to the consensus of the IWGDF [17]. Infected ulcers were clinically diagnosed by the presence of at least 2 signs or symptoms of inflammation (i.e., redness, warmth, induration, and pain/tenderness), or purulent secretions. Furthermore, systemic inflammatory findings (i.e., fever, leukocytosis, and C reactive protein) were also assessed to classify the severity of the infection [22]. All study participants signed a written informed consent form before inclusion in the study. The study was conducted in accordance with the Declaration of Helsinki, and the protocol was approved by the Ethics Committee of the University Hospital Arnau de Vilanova.

### 2.2. Instruments

#### 2.2.1. Diabetic Foot Ulcer Scale-Short Form (DFS-SF)

The DFS-SF is a disease-specific questionnaire that assesses the impact of DFU on the QoL [13]. This instrument contains 29 items based on the following 6 subscales: leisure, physical health, dependence/daily life, negative emotions, worried about ulcers/feet, and bothered by ulcer care. The score of each subscale is calculated based on a scale from 0 (poorer QoL) to 100 (higher QoL). This was validated in reference to the original extended questionnaire DFS that contains 58 items and 11 domains [12].

#### 2.2.2. 36-Item Short-Form Health Survey (SF-36)

The SF-36 is a generic instrument that assesses the health status of the patients [23]. It is composed of 36 items that are grouped into 8 subscales: physical functioning, physical role, bodily pain, general health, vitality, social functioning, emotional role, and mental health. These subscales are weighted into physical and mental health summary scores. Each subscale is scored with a range from 0 to 100 points and is normalized using US norms; higher scores indicate a better health-related quality of life (HRQoL). 

#### 2.2.3. EuroQoL 5D Health Utility Index (EQ-5D)

EQ-5D is a generic questionnaire designed for use in different types of health states and diseases, as well as in the general populations of several countries [24]. This instrument includes 5 dimensions: mobility, self-care, usual activities, pain/discomfort, and anxiety/depression. Each item is divided into 3 levels: no problems, some problems, and extreme problems. A single numeric index of health status (EQ-5D index value) is defined by combining the 5 dimensions using UK weights. The questionnaire also includes a visual analogue scale (VAS) where participants are asked to indicate how they rate their current health status on a scale from 0 (minimum) to 100 points (maximum or the best imaginable health state).

### 2.3. Transcultural Adaptation of the DFS-SF

First, the English version was translated into Spanish by two independent translators who were experts in both languages. The 2 translated versions were later compared, and noted differences were discussed by a group of patients and a group of health-care professionals with expertise in diabetic foot disease. After this, the first Spanish version was approved and back-translated by a third English native speaker. Researchers then compared this back-translation and the original English questionnaire. The final Spanish version was assessed by the group of experts to determine its content validity using a Likert scale (File S1; available at https://www.irblleida.org/media/upload/arxius/VARIS/File%20S1_cuestionario.pdf).

### 2.4. Sample Size

The measure of internal consistency was determined by accepting an alpha risk of 0.05 and a beta risk of 0.2 in a two-sided test. We calculated that a sample of 124 patients would be required to designate a Cronbach’s α coefficient ≥ 0.3 as statistically significant. Anticipating a maximum dropout rate of 15%, the final required sample size resulted in 143 subjects.

### 2.5. Data Analysis

Data were described by using mean and standard deviation for quantitative variables and absolute and relative frequencies for qualitative variables.

The reliability was measured through internal consistency and reproducibility assessment. The internal consistency was measured by Cronbach’s α coefficient [25]. According to the standard protocol, the Cronbach value should be ≥0.70. The reproducibility was estimated using the intraclass correlation coefficient (ICC) defined by a single rater two-way mixed-effects model for quantitative variables. Baseline and first follow-up visit (at 7 days after) ratings were compared, assuming no changes for unhealed ulcers.

The validity was measured through a criterion and construct validity assessment. The criterion validity was only determined according to the concurrent validity in reference to the generic questionnaires by measuring the Pearson’s correlation coefficient of the scores of the DFS-SF subscales and comparing them with the ones of the corresponding SF-36 and EQ-5D subscales. Their values should be >0.3, which is indicative of a moderate correlation. The construct validity was assessed through a confirmatory factor analysis of the DFS-SF to test the questionnaire structure. The comparative fit index (CFI), the root mean square error of approximation (RMSEA), and the standardized root mean square residual (SRMR) were estimated. Their values should be ≥0.95, ≤0.06, and ≤0.08, respectively, as indicative of a good fit to the subscales structure.

The sensitivity to changes over time is defined as the ability of an instrument to measure a change in the state regardless of whether the change is relevant or meaningful to the decision-maker [26,27]. This was assessed through the smoothed trends from baseline until the last visit and, depending on the healing state of the ulcer, at the last available visit; changes between healed and non-healed patients were compared using the Mann–Whitney test. All analyses were performed with the R software with a significance level of 0.05 [28].

## 3. Results

The characteristics of the 141 participants are shown in Table 1. A high frequency of patients with neuropathy (92.9%) and macrovascular complications (89.4%) was observed. At the end of the study, 107 of the patients (75.8%) had experienced healing (Table S1).

The content validity was assessed by seven experts who rendered an average score of 3.46 (86% of the maximum score of 4). The internal consistency of the DFS-SF subscales was good (Cronbach’s α range = 0.720–0.948) (Table 2). The internal consistency of the subscales was not improved or marginally improved by item deletion. The DFS-SF domains showed reproducibility measures that ranged from good to excellent (ICC estimates = 0.77–0.92).

The DFS-SF subscales were correlated with SF-36 and EQ-5D subscales (Table 3). The Leisure and Worried about ulcers/feet subscales were moderately correlated with Social functioning (*r* > 0.32, *p* < 0.001) of the SF-36. Physical health was moderately correlated with Physical functioning, Role, and Component summary as well as Bodily pain, Social functioning, and Vitality of the SF-36 and with the EQ-5D score (*r* > 0.32, *p* < 0.001). Dependence was largely correlated with the Physical functioning, Role physical, and Overall physical components (*r* > 0.55, *p* < 0.001); also, Dependence was moderately correlated with Social functioning, Vitality, and Bodily pain of the SF-36 (*r* > 0.4, *p* < 0.001) and with the EQ-5D index value (*r* = 0.45, *p* < 0.001). The Negative emotions subscale was largely correlated with Social functioning (*r* > 0.52, *p* < 0.001) and all other SF-36 domains, the EQ-5D score, and VAS. Bothered by ulcer care was moderately correlated with Physical functioning, Role, and Component summary as well as Vitality and Social functioning (*r* > 0.33, *p* < 0.001).

**Figure 1:**
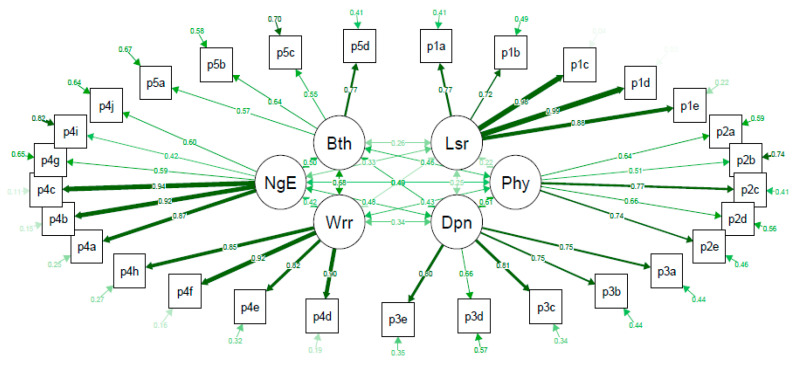
Confirmatory factor analysis of the different subscales of the Diabetes Foot Ulcer Scale-Short Form (DFS-SF). Bth, bothered by ulcer care; NgE, negative emotions; Wrr, worried about ulcers/feet; Lsr, leisure; Phy, physical health; Dpn, dependence/daily life. p1a–p5d are the items per subscale of the DFS-SF questionnaire. Each arrow between the questionnaire items and the subscale that they are measuring shows the standardized pattern coefficients for this relationship, where values closer to 1.0 (wider and darker) are indicative of better fit, and the circled arrow represented in each questionnaire item shows the residuals. The arrows connecting subscales show the pairwise correlation between them. Comparative fit index (CFI) = 0.844 (stands for comparative fit index and a value of ≥0.95 is indicative of good fit); root mean square error of approximation (RMSEA) = 0.096 (stands for the root mean square error of approximation and a value of ≤0.06 is indicative of acceptable model fit); standardized root mean square residual (SRMR) = 0.094 (stands for standardized root mean square residual and a value of ≤0.08 is indicative of an acceptable model).

**Figure 2:**
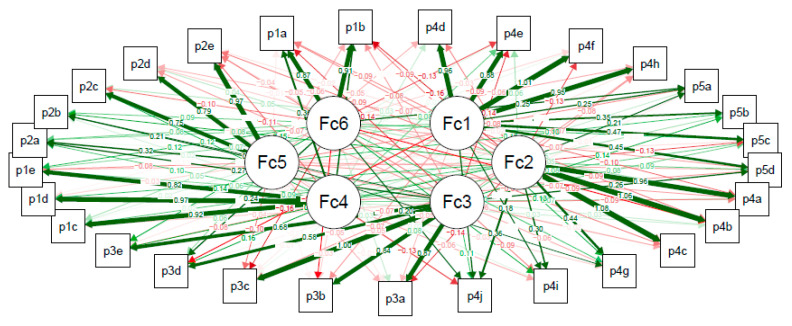
Exploratory factor analysis of the different subscales of the Diabetes Foot Ulcer Scale-Short Form (DFS-SF). Fc1–Fc6, Factor 1 to Factor 6 of the exploratory factor analysis. p1a–p5d are the items per subscale of the DFS-SF questionnaire. Maximum likelihood exploratory factor analysis fixing the total number of subscales in 6 with a promax rotation. Each arrow shows the loading of each questionnaire item to each subscale, where the width and color intensity of each arrow is proportional to each value. Loadings closer to 1.0 in absolute value are indicative of stronger relationships with subscales (red color is indicative of opposite relationship). The explained cumulative variance by the 6 factors was 65.5 %.

The confirmatory factor analysis of the DFS-SF showed that the questionnaire structure was not validated (Figure 1). The full set of data corresponding to Figure 1 is provided in Table S2. CFI, RMSEA, and SRMR were 0.844, 0.095, and 0.093, respectively, showing a lack of good fit to the questionnaire structure. The exploratory factor analysis of 6 factors divided the Leisure subscale into 2 subscales (questions p1a-p1b refer to hobbies and leisure activities vs. p5c-p1e that refer to holidays, weekends, and activities planning), and did not identify the Bothered by ulcer care subscale but joined all its items to the Dependence or Daily life subscale (Figure 2). The full set of data corresponding to Figure 2 is provided in Table S3.

Finally, the DFS-SF was sensitive to the ulcer status changes (*p* < 0.001) (Table 4). Healed patients had significantly higher punctuations in all subscales in comparison with the unhealed group.

## 4. Discussion

This is the first study designed to assess the psychometric properties of a specific questionnaire in patients with DFU in Spain. Our results showed that the Spanish DFS-SF had good internal consistency and criterion validity with excellent reproducibility. Although its structure was not validated, the sensitivity to ulcer changes over time was high. The internal consistency of the DFS-SF subscales was good, with a Cronbach’s α range similar to that of the original English version [13]. Furthermore, reproducibility was excellent, as observed in the English version [12,13].

The Spanish DFS-SF demonstrated good criterion validity; the overall physical and mental components of the SF-36 had moderate correlations with each of the physical and mental subscales of the DFS-SF. This is similar to the English, Greek, and Chinese studies [13,14,15]; however, the Polish version did not identify any correlation in the physical subscales [16].

Moreover, the Spanish DFS-SF demonstrated poor construct validity, which is in contrast with previously published studies [13,14,15,16]. This could be due to the cultural differences between the English and Spanish populations; in the Spanish population, the variables are distinctively distributed and grouped in comparison with other countries.

The Spanish DFS-SF showed sensitivity to ulcer changes over time as observed in the versions in other languages [7,13,16]. Furthermore, the DFS-SF is more sensitive than the generic instruments because in the latter case the impact of other conditions on the QoL may be confounded [14,16,29]. This is mainly due to the extensive use of generic instruments to measure QoL in many previous studies. Therefore, this is the first specific validated questionnaire to assess the QoL of subjects with diabetes and foot ulcers in Spain.

As mentioned previously, the optimal assessment of QoL for specific conditions, such as DFU, requires the use of instruments adequately designed for this purpose [10]. Apart from its original English version, the DFS questionnaire is currently available only in Chinese, Greek, and Polish [14,15,16]. We strongly believe that there is a great need to provide specific tools for measuring the QoL in subjects with DFU in many other countries and languages. The absence of a proper tool for this purpose in Spain was the main reason leading us to develop the current work. Fortunately, we are hereby providing the DFS-SF validated in Spain. Further, the current version uses the Spanish language, which is common to hundreds of millions worldwide, especially in South and Central America. However, we think that the current version may not be used straightforwardly in other Spanish speaking communities without a proper cultural adaptation.

This study has some limitations. Firstly, the QoL measurements could be influenced by the comorbidities present in these patients. Secondly, the test–retest was performed 7 days after treatment was started, and this may affect the reproducibility; for ethical reasons, all patients received treatment from baseline. However, this study has several strengths. We validated the DFS-SF and compared it with that of the EQ-5D, which confers more quality and precision in the validation process [13,14,15,16]. Additionally, this study reports good psychometric properties of the only diabetes-specific questionnaire of QoL used in Spanish patients with DFU. Furthermore, the prospective design allowed assessing the sensitivity to changes regarding the impact of DFU on the QoL.

## 5. Conclusions

In conclusion, the current version of the DFS-SF in Spanish has good psychometric properties despite the construct validity not being optimal. This questionnaire is a sensitive tool with good performance to capture ulcer changes over time. Finally, the Spanish version of the DFS-SF can be readily available for assessing the QoL in subjects with DFU in Spain. Further, this tool will be useful in clinical and research settings.

## Figures and Tables

**Table 1:** Clinical and sociodemographic characteristics of the study group at baseline.

Characteristics	Study Group (*n* = 141)
Age (years)	68.3 (13.3)
Male (sex)	95 (67.4)
Race (Caucasian)	140 (99.3)
Educational level	
Not even primary	57 (40.4)
Complete primary	47 (33.3)
Secondary high cycle	28 (19.9)
Graduate or higher	9 (6.4)
Employed	24 (17.0)
Smoking	
Never	63 (44.7)
Current or former	78 (55.4)
Type 2 diabetes	134 (95.0)
BMI (kg/m^2^)	29.0 (4.9)
HbA1c (%)	7.5 (1.6)
Hypertension	116 (82.3)
Dyslipidemia	87 (61.7)
Microvascular complications	
Retinopathy	96 (68.1)
Nephropathy	51 (36.2)
Neuropathy	131 (92.9)
Cardiovascular disease ^1^	126 (89.4)
Diabetes therapy	
OAD	41 (29.1)
OAD + insulin	57 (40.4)
Insulin	36 (25.5)
Diet	7 (5.0)
Antiplatelet agents	94 (66.7)
Dialysis	8 (5.7)
Ulcer type	
Neuropathic	87 (61.7)
Ischemic	9 (6.4)
Neuroischemic	45 (31.9)
Infected ulcer	83 (58.9)
Previous amputation	
Minor	41 (29.1)
Major	2 (1.4)
Charcot foot disease	9 (6.4)

Data are shown as mean (SD) for quantitative variables or n (%) for qualitative variables. ^1^ Cardiovascular disease included cerebrovascular disease, peripheral artery disease and ischemic heart disease. BMI, body mass index; HbA1c, glycated hemoglobin; OAD, oral antidiabetic agents.

**Table 2:** Inter-item internal consistency and reproducibility of the Diabetes Foot Ulcer-Short Form (DFS-SF) subscales.

Dfs-Sf Subscales	Number of Items ^1^	Range of Correlations ^2^	Average Inter-Item Correlation	Cronbach’s Alpha	Reproducibility (ICC [95%CI])
Leisure	5	0.678–0.966	0.786	0.948	0.87 [0.82, 0.91]
Physical health	5	0.195–0.641	0.433	0.792 ^3^	0.78 [0.70, 0.84]
Worried about ulcers/feet	4	0.678–0.818	0.760	0.927	0.92 [0.89, 0.94]
Dependence/daily life	5	0.417–0.662	0.566	0.867	0.77 [0.68, 0.83]
Negative emotions	6	0.310–0.886	0.553	0.881 ^4^	0.84 [0.79, 0.89]
Bothered by ulcer care	4	0.304–0.537	0.391	0.720	0.77 [0.69, 0.83]

^1^ Number of items per subscale. ^2^ Inter-item Pearson’s correlations. ^3^ Improved from 0.792 to 0.794 if the second item for this subscale is deleted. ^4^ Improved from 0.881 to 0.892 if the fifth item for this subscale is deleted. Reproducibility was estimated by assuming no changes between the baseline and first visit (at 7 days from baseline) for unhealed ulcers. ICC, intraclass correlation coefficients; CI, confidence interval.

**Table 3:** Criterion validity of the Diabetic Foot Ulcer Scale-Short Form by Pearson’s correlation coefficients with SF-36 and EQ-5D overall and subscale scores.

Domains.	DFS-SF Subscales					
	Leisure	Physical Health	Dependence/Daily Life	Worried about Ulcers/Feet	Negative Emotions	Bothered by Ulcer Care
SF-36 subscales						
Physical functioning	0.052	0.473 **	0.737 **	0.220 *	0.398 **	0.370 **
Role physical	0.135	0.413 **	0.558 **	0.258 *	0.445 **	0.376 **
Bodily pain	0.116	0.438 **	0.403 **	0.224 *	0.395 **	0.277 *
General health	0.057	0.165	0.139	0.183 *	0.350 **	0.198 *
Vitality	0.126	0.327 **	0.418 **	0.245 *	0.383 **	0.345 **
Social functioning	0.358 **	0.401 **	0.453 **	0.321 **	0.526 **	0.336 **
Role emotional	0.140	0.271 **	0.221 *	0.187 *	0.365 **	0.288 *
Mental health	0.079	0.204 *	0.174 *	0.256 *	0.484 **	0.199 *
Overall physical component ^1^	0.082	0.482 **	0.674 **	0.229 *	0.394 **	0.368 **
Overall mental component ^2^	0.178 *	0.195 *	0.110	0.248 *	0.449 **	0.235 *
EQ-5D subscales						
VAS	0.062	0.166 *	0.223 *	0.240 *	0.306 **	0.183 *
EQ-5D index value	0.039	0.445 **	0.454 **	0.204 *	0.324 **	0.184 *

^1,2^ Calculated according to the SF-36 subscales involved physical and mental roles. VAS, visual analog scale. * *p* < 0.05; ** *p* < 0.001.

**Table 4:** Summary of the descriptive analysis of sensitivity to change assessment of Diabetes Foot Ulcer Scale-Short Form (DFS-SF) subscales according to healed/unhealed group.

Variables	Unhealed ^1^ (*n* = 34)	Healed (*n* = 107)	*p*-Overall
Change in DFS Leisure from baseline	0.00 [0.00; 8.75]	5.00 [0.00; 25.00]	0.014
Change in DFS Physical Health from baseline	0.00 [−10.00; 0.00]	20.00 [5.00; 30.00]	<0.001
Change in DFS Dependence from baseline	0.00 [0.00; 3.75]	10.00 [0.00; 25.00]	<0.001
Change in DFS Negative Emotions from baseline	0.00 [0.00; 4.17]	8.34 [0.00; 25.00]	0.001
Change in DFS Worried about ulcers/feet from baseline	0.00 [0.00; 18.80]	31.2 [9.38; 50.00]	<0.001
Change in DFS Bothered by ulcer care from baseline	0.00 [0.00; 6.25]	18.80 [0.00; 37.50]	<0.001

Data are shown as median [confidence interval]. ^1^ We are including here all subjects that remained unhealed until the last visit, until they were lost to follow-up or experienced an unfavorable event (amputation, death).

## Supplementary Materials

The following are available online at https://www.mdpi.com/2077-0383/9/8/2497/s1. File S1: Cuestionario Diabetic Foot Ulcer Scale-Short Form (DFS-SF). Table S1: Frequency of healed ulcers, amputations, and deaths of the follow-up visits. Table S2: Confirmatory factor analysis of the different subscales of the Diabetes Foot Ulcer Scale-Short Form (DFS-SF). Table S3: Exploratory factor analysis of the different subscales of the Diabetes Foot Ulcer Scale-Short Form (DFS-SF).
