# Correction: Martinez-Gonzalez et al. Adaptation and Validation of the Diabetic Foot Ulcer Scale-Short Form in Spanish Subjects. *J. Clin. Med.* 2020, *9*, 2497

**DOI:** 10.3390/jcm11195963

**Published:** 2022-10-10

**Authors:** Dolores Martinez-Gonzalez, Montserrat Dòria, Montserrat Martínez-Alonso, Nuria Alcubierre, Joan Valls, José Verdú-Soriano, Minerva Granado-Casas, Didac Mauricio

**Affiliations:** 1 Lleida Institute for Biomedical Research Dr. Pifarré Foundation IRBLleida, University of Lleida, Rovira Roure, 80, 25198 Lleida, Spain; 2 Department of Endocrinology and Nutrition, Health Sciences Research Institute and University Hospital Germans Trias i Pujol, Camí de les Escoles S/N, 08916 Badalona, Spain; 3 Diabetic Foot Unit, University Hospital Arnau de Vilanova, Rovira Roure 80, 25198 Lleida, Spain; 4 Systems Biology and Statistical Methods for Biomedical Research, IRBLleida, University of Lleida, Rovira Roure 80, 25198 Lleida, Spain; 5 Department of Community Nursing, Preventive Medicine and Public Health and History of Science, University of Alicante, Carretera de Sant Vicent del Raspeig s/n, 03080 Alicante, Spain; 6 Grupo Nacional de Estudio y Asesoramiento de Úlceras por Presión (GNEAUPP) Steering Committee, 26004 Logroño, Spain; 7 Centre for Biomedical Research on Diabetes and Associated Metabolic Diseases (CIBERDEM), Instituto de Salud Carlos III, 08041 Barcelona, Spain; 8 Department of Endocrinology & Nutrition, Hospital de la Santa Creu i Sant Pau, Sant Quintí, 89, 08041 Barcelona, Spain; 9 Faculty of Medicine, University of Vic (UVIC/UCC), 08500 Vic, Spain

## Error in Authorship

In the original publication [1], there was a mistake in Authorship as published. The second author’s name should be “Montserrat Dòria”, instead of “Montse Dòria”. The corrected Authorship has been updated.

## Missing Funding

In the original publication [1], the funder This project was developed within the framework of the Doctorate in the Department of Medicine, Autonomous University of Barcelona was not included. The corrected Funding appears below. The authors apologize for any inconvenience caused and state that the scientific conclusions are unaffected. This correction was approved by the Academic Editor. The original publication has also been updated.

**Funding:** CIBERDEM is an initiative from Instituto de Salud Carlos III (Plan Nacional de I + D + I and Fondo Europeo de Desarrollo Regional). M.G.-C. held a predoctoral fellowship from the Ministerio de Educación, Cultura y Deporte, FPU15/03005. This project was developed within the framework of the Doctorate in the Department of Medicine, Autonomous University of Barcelona.
