# Supplementary material for: Adaptation and Validation of the Diabetic Foot Ulcer Scale-Short Form in Spanish Subjects
Source: J Clin Med. 2020 Aug 3;9(8):2497. doi: 10.3390/jcm9082497 (PMC7465700; doi:10.3390/jcm9082497)
Supplement: Supplementary file 1 [file jcm-09-02497-s001.zip › File S1_cuestionario.pdf]

Cuestionario Diabetic Foot Ulcer Scale-Short Form (DFS-SF)

1. ¿Cómo le han afectado sus problemas de la úlcera en el pie?

a) Le han impedido practicar sus aficiones y actividades de ocio:

1= en absoluto
2= un poco
3= moderadamente
4= bastante
5= mucho

b) Le han cambiado el tipo de aficiones y actividades de ocio:

1= en absoluto
2= un poco
3= moderadamente
4= bastante
5= mucho

c) Le han impedido ir de vacaciones o hacer una salida de fin de semana:

1= en absoluto
2= un poco
3= moderadamente
4= bastante
5= mucho

d) Le han modificado ir de vacaciones o hacer una salida de fin de semana:

1= en absoluto
2= un poco
3= moderadamente
4= bastante
5= mucho

e) Le han hecho pasar más tiempo planeando y organizando sus actividades:

1= en absoluto
2= un poco
3= moderadamente
4= bastante
5= mucho

2. Debido a sus problemas de úlcera de pie, ¿con qué frecuencia se ha sentido...

a) cansado o fatigado?

1= nunca
2= casi nunca
3= algunas veces
4= casi siempre
5= siempre

© 2020 Institut de Recerca Biomèdica de Lleida Fundació Dr. Pifarré (IRBLleida). Avda Alcalde Rovira Roure, 80, 25198, Lleida (España). https://www.irblleida.org. Esta obra está bajo una licencia de Creative Commons Reconocimiento 4.0 Internacional.

b) agotado?

1= nunca
2= casi nunca
3= algunas veces
4= casi siempre
5= siempre

c) con dificultades para dormir?

1= nunca
2= casi nunca
3= algunas veces
4= casi siempre
5= siempre

d) con dolor al caminar o estar de pie?

1= nunca
2= casi nunca
3= algunas veces
4= casi siempre
5= siempre

e) con dolor durante la noche?

1= nunca
2= casi nunca
3= algunas veces
4= casi siempre
5= siempre

3. Debido a sus problemas de úlcera de pie, ¿con qué frecuencia ha...

a) dependido de otras personas que le ayuden en su cuidado personal?

1= nunca
2= casi nunca
3= algunas veces
4= casi siempre
5= siempre

b) dependido de otras personas para realizar labores rutinarias del hogar?

1= nunca
2= casi nunca
3= algunas veces
4= casi siempre
5= siempre

c) dependido de otras personas para salir de casa?

1= nunca
2= casi nunca
3= algunas veces
4= casi siempre

© 2020 Institut de Recerca Biomèdica de Lleida Fundació Dr. Pifarré (IRBLleida). Avda Alcalde Rovira Roure, 80, 25198, Lleida (España). https://www.irblleida.org. Esta obra está bajo una licencia de Creative Commons Reconocimiento 4.0 Internacional.

5= siempre

d) dedicado más tiempo en planificar y organizar su vida diaria?

1= nunca
2= casi nunca
3= algunas veces
4= casi siempre
5= siempre

e) sentido que para hacer cualquier actividad tardaba más tiempo de lo que le hubiera gustado?

1= nunca
2= casi nunca
3= algunas veces
4= casi siempre
5= siempre

4. Debido a sus problemas de úlcera de pie, ¿se ha sentido...

a) enfadado porqué no ha podido hacer lo que a usted le gustaría?

1= en absoluto
2= un poco
3= moderadamente
4= bastante
5= mucho

b) frustrado porqué otros han tenido que hacer algo que a usted le hubiera gustado hacer?

1= en absoluto
2= un poco
3= moderadamente
4= bastante
5= mucho

c) frustrado porqué no ha podido hacer lo que le hubiera gustado?

1= en absoluto
2= un poco
3= moderadamente
4= bastante
5= mucho

d) preocupado por si su úlcera(s) no se curará(n) nunca?

1= en absoluto
2= un poco
3= moderadamente
4= bastante
5= mucho

e) preocupado por si pudiera sufrir una amputación?

© 2020 Institut de Recerca Biomèdica de Lleida Fundació Dr. Pifarré (IRBLleida). Avda Alcalde Rovira Roure, 80, 25198, Lleida (España). https://www.irblleida.org. Esta obra está bajo una licencia de Creative Commons Reconocimiento 4.0 Internacional.

1= en absoluto
2= un poco
3= moderadamente
4= bastante
5= mucho

f) preocupado por una posible herida en los pies?

1= en absoluto
2= un poco
3= moderadamente
4= bastante
5= mucho

g) deprimido porqué no ha podido hacer lo que le ha gustado?

1= en absoluto
2= un poco
3= moderadamente
4= bastante
5= mucho

h) preocupado por la aparición de nuevas úlceras en el futuro?

1= en absoluto
2= un poco
3= moderadamente
4= bastante
5= mucho

i) enfadado porqué esto le ha pasado a usted?

1= en absoluto
2= un poco
3= moderadamente
4= bastante
5= mucho

j) frustrado porqué tiene problemas para desplazarse?

1= en absoluto
2= un poco
3= moderadamente
4= bastante
5= mucho

5. Debido a su problema de úlcera en el pie, ¿con qué frecuencia se ha sentido molesto por...

a) tener que mantener la úlcera del pie sin tener que cargar peso sobre ella?

1= nunca
2= casi nunca
3= algunas veces
4= casi siempre
5= siempre

© 2020 Institut de Recerca Biomèdica de Lleida Fundació Dr. Pifarré (IRBLleida). Avda Alcalde Rovira Roure, 80, 25198, Lleida (España). https://www.irblleida.org. Esta obra está bajo una licencia de Creative Commons Reconocimiento 4.0 Internacional.

b) la cantidad de tiempo que implica el cuidado de la úlcera del pie?

1= nunca
2= casi nunca
3= algunas veces
4= casi siempre
5= siempre

c) el aspecto, olor o supuración de la úlcera?

1= nunca
2= casi nunca
3= algunas veces
4= casi siempre
5= siempre

d) tener que depender de otros para ayudarle a curar la úlcera?

1= nunca
2= casi nunca
3= algunas veces
4= casi siempre
5= siempre

Puntuación total:

© 2020 Institut de Recerca Biomèdica de Lleida Fundació Dr. Pifarré (IRBLleida). Avda Alcalde Rovira Roure, 80, 25198, Lleida (España). https://www.irblleida.org. Esta obra está bajo una licencia de Creative Commons Reconocimiento 4.0 Internacional.
