# Supplementary material for: Adaptation and Validation of the Diabetic Foot Ulcer Scale-Short Form in Spanish Subjects
Source: J Clin Med. 2020 Aug 3;9(8):2497. doi: 10.3390/jcm9082497 (PMC7465700; doi:10.3390/jcm9082497)
Supplement: Supplementary file 1 [file jcm-09-02497-s001.zip › Table S1_rev.docx]

**Table S1.** Frequency of healed ulcers, amputations and deaths of the follow-up visits

| **Visits** | **Healed** | **Unhealed** | **Minor amputation** | **Major amputation** | **Follow-up losses** | **Death** |
| --- | --- | --- | --- | --- | --- | --- |
| Visit 2 | 3 | 128 | 3 | 0 | 7 | 0 |
| Visit 3 | 73 | 46 | 2 | 0 | 7 | 0 |
| Visit 4 | 22 | 17 | 1 | 0 | 5 | 1 |
| Visit 5 | 9 | 5 | 0 | 3 | 0 | 0 |

Data are shown as absolute frequencies. Visits 2, 3, 4 and 5 were performed at 7 days, 4 weeks, 12 weeks and 26 weeks from baseline, respectively.
