# Supplementary material for: Adaptation and Validation of the Diabetic Foot Ulcer Scale-Short Form in Spanish Subjects
Source: J Clin Med. 2020 Aug 3;9(8):2497. doi: 10.3390/jcm9082497 (PMC7465700; doi:10.3390/jcm9082497)
Supplement: Supplementary file 1 [file jcm-09-02497-s001.zip › Table S2.docx]

**Table S2.** Confirmatory factor analysis of the different subscales of the Diabetes Foot Ulcer Scale-Short Form (DFS-SF)

| **Subscale** | **Item** | **Standardized coefficient** | **se** | **z** |
| --- | --- | --- | --- | --- |
| Leisure | p1a | 0.765 | 0.036 | 21.474 |
|  | p1b | 0.717 | 0.042 | 17.217 |
|  | p1c | 0.978 | 0.006 | 161.891 |
|  | p1d | 0.986 | 0.005 | 186.162 |
|  | p1e | 0.885 | 0.019 | 46.468 |
| Physical health | p2a | 0.638 | 0.059 | 10.735 |
|  | p2b | 0.505 | 0.071 | 7.084 |
|  | p2c | 0.770 | 0.047 | 16.555 |
|  | p2d | 0.665 | 0.057 | 11.689 |
|  | p2e | 0.735 | 0.050 | 14.739 |
| Dependence/daily life | p3a | 0.749 | 0.044 | 17.169 |
|  | p3b | 0.747 | 0.044 | 17.169 |
|  | p3c | 0.810 | 0.037 | 17.005 |
|  | p3d | 0.656 | 0.054 | 22.129 |
|  | p3e | 0.804 | 0.037 | 21.504 |
| Worried about ulcer/feet | p4d | 0.898 | 0.021 | 43.025 |
|  | p4e | 0.823 | 0.031 | 26.825 |
|  | p4f | 0.916 | 0.019 | 48.840 |
|  | p4h | 0.855 | 0.026 | 32.262 |
| Negative emotions | p4a | 0.868 | 0.024 | 36.400 |
|  | p4b | 0.919 | 0.017 | 53.397 |
|  | p4c | 0.944 | 0.015 | 65.102 |
|  | p4g | 0.595 | 0.057 | 10.467 |
|  | p4i | 0.425 | 0.071 | 5.959 |
|  | p4j | 0.597 | 0.057 | 10.562 |
| Bothered by ulcer care | p5a | 0.572 | 0.068 | 8.432 |
|  | p5b | 0.644 | 0.061 | 10.481 |
|  | p5c | 0.552 | 0.070 | 7.941 |
|  | p5d | 0.768 | 0.051 | 15.019 |

p1a – p5d are the items per subscale of the DFS-SF questionnaire. Values closer to 1.0 are indicative of better fit. Comparative fit index (CFI) = 0.844 (stands for comparative fit index and a value of ≥ 0.95 is indicative of good fit); root mean square error of approximation (RMSEA) = 0.096 (stands for the root mean square error of approximation and a value of ≤ 0.06 is indicative of acceptable model fit); standardized root mean square residual (SRMR) = 0.094 (stands for standardized root mean square residual and a value of ≤ 0.08 is indicative of an acceptable model).
