# Supplementary material for: Adaptation and Validation of the Diabetic Foot Ulcer Scale-Short Form in Spanish Subjects
Source: J Clin Med. 2020 Aug 3;9(8):2497. doi: 10.3390/jcm9082497 (PMC7465700; doi:10.3390/jcm9082497)
Supplement: Supplementary file 1 [file jcm-09-02497-s001.zip › Table S3.docx]

**Table S3.** Exploratory factor analysis of the different subscales of the Diabetes Foot Ulcer Scale-Short Form (DFS-SF)

| **Items** | **Factor 1** | **Factor 2** | **Factor 3** | **Factor 4** | **Factor 5** | **Factor 6** |
| --- | --- | --- | --- | --- | --- | --- |
| p1a | -0.085 | -0.082 | -0.144 | 0.358 | -0.013 | 0.868 |
| p1b | -0.093 | -0.162 | -0.071 | 0.305 | -0.056 | 0.906 |
| p1c | 0.006 | 0.093 | -0.037 | 0.918 | 0.023 | 0.052 |
| p1d | 0.005 | 0.109 | 0.004 | 0.972 | -0.023 | 0.052 |
| p1e | 0.069 | -0.053 | 0.064 | 0.817 | -0.078 | 0.121 |
| p2a | 0.078 | 0.009 | 0.269 | 0.096 | 0.315 | 0.083 |
| p2b | 0.003 | 0.147 | 0.180 | 0.024 | 0.207 | 0.087 |
| p2c | 0.045 | 0.004 | 0.004 | 0.119 | 0.752 | -0.098 |
| p2d | -0.053 | -0.037 | -0.070 | -0.024 | 0.788 | 0.041 |
| p2e | -0.020 | -0.091 | -0.087 | -0.112 | 0.967 | -0.041 |
| p3a | -0.047 | -0.136 | 0.869 | -0.014 | -0.052 | 0.034 |
| p3b | -0.072 | 0.076 | 0.841 | -0.032 | -0.009 | -0.220 |
| p3c | 0.010 | -0.071 | 0.995 | -0.012 | -0.100 | -0.164 |
| p3d | -0.186 | -0.008 | 0.577 | 0.159 | 0.061 | 0.241 |
| p3e | -0.017 | 0.149 | 0.678 | -0.084 | 0.020 | 0.144 |
| p4a | -0.104 | 0.958 | -0.089 | 0.073 | -0.069 | 0.049 |
| p4b | -0.091 | 1.064 | 0.033 | 0.031 | -0.001 | -0.211 |
| p4c | -0.095 | 1.079 | -0.018 | 0.004 | -0.062 | -0.090 |
| p4d | 0.958 | -0.089 | 0.005 | 0.034 | -0.052 | -0.005 |
| p4e | 0.884 | 0.058 | -0.140 | 0.080 | 0.017 | -0.126 |
| p4f | 1.005 | -0.128 | -0.003 | -0.064 | 0.011 | -0.030 |
| p4g | 0.133 | 0.440 | -0.058 | -0.032 | 0.172 | 0.101 |
| p4h | 0.949 | -0.077 | -0.067 | -0.017 | 0.005 | -0.062 |
| p4i | 0.184 | 0.304 | -0.090 | -0.059 | 0.019 | 0.141 |
| p4j | 0.236 | 0.363 | 0.111 | -0.129 | -0.051 | 0.204 |
| p5a | 0.252 | 0.209 | -0.014 | 0.033 | -0.082 | 0.249 |
| p5b | 0.346 | -0.006 | 0.136 | 0.052 | 0.105 | 0.004 |
| p5c | 0.467 | -0.130 | 0.080 | 0.084 | -0.023 | 0.104 |
| p5d | 0.447 | 0.087 | 0.262 | -0.074 | -0.064 | -0.072 |

p1a – p5d are the items per subscale of the DFS-SF questionnaire. Maximum likelihood exploratory factor analysis fixing the total number of subscales in 6 with a promax rotation. Loadings closer to 1.0 in absolute value are indicative of stronger relationships with subscales; negative values are an opposite relationship. The explained cumulative variance by the 6 factors was 65.5 %.
